# Impact of modified SSD soft brace on surgical outcomes and wound healing duration following hand scar contracture release in RDEB patients

**DOI:** 10.3389/fsurg.2026.1877597

**Published:** 2026-07-23

**Authors:** Teng Guo, Chong Wu, Xinhe Jiao

**Affiliations:** 1 Department of Plastic Surgery, The Second Affiliated Hospital of Zhengzhou University, Zhengzhou, Henan, China; 2 The Fifth Clinical Medical College of Henan University of Chinese Medicine (Zhengzhou People’s Hospital), Zhengzhou, Henan, China

**Keywords:** hand scar contracture, home compliance, recessive dystrophic epidermolysis bullosa, silver sulfadiazine, soft brace

## Abstract

**Objective:**

To investigate the clinical efficacy of a modified soft brace incorporating a Silver Sulfadiazine (SSD) oil-based dressing in improving wound healing and home care quality following hand scar contracture release surgery in patients with RDEB.

**Methods:**

Thirty-five RDEB patients (aged 8–21 years) who underwent hand contracture release between 2017 and 2023 were assigned to Traditional (Vaseline gauze + brace, *n* = 17) or Modified (SSD dressing + brace, *n* = 18) groups. Perioperative outcomes, wound healing duration, compliance (RHCS), and recurrence-free survival were compared.

**Results:**

Baseline characteristics were comparable. Compared to the Traditional Group, the Modified Group had significantly fewer dressing changes (2.33 ± 0.49 vs. 4.18 ± 0.73, *P* < 0.001), shorter healing duration (23.33 ± 3.40 vs. 36.24 ± 5.09 days, *P* < 0.001), lower epithelial stripping rate (11.1% vs. 64.7%, *P* = 1.6E-3), and no refractory wounds. The Modified Group also showed higher RHCS total scores (84.94 ± 9.62 vs. 46.12 ± 19.12, *P* < 0.001) and fewer spontaneous nocturnal blisters (11.1% vs. 64.7%). Univariable Cox regression indicated the modified regimen was associated with delayed recurrence. Kaplan–Meier analysis showed delayed recurrence in the Modified Group by log-rank test (*P* = 0.03), and univariable Cox analysis yielded HR 0.39, 95% CI 0.16–0.95, albeit with wide confidence intervals and limited late-term power.

**Conclusion:**

In this retrospective cohort, the SSD-containing non-adherent dressing used with a soft brace was associated with fewer dressing changes, less dressing-related trauma, and better reported tolerance. Prospective controlled studies with validated functional and compliance outcomes are required.

## 1 Introduction

Recessive Dystrophic Epidermolysis Bullosa (RDEB) is a severe genetic dermatosis caused by biallelic mutations in the *COL7A1*gene. The resultant deficiency of type VII collagen disrupts the structural integrity of the dermo-epidermal junction, rendering the skin susceptible to blistering, erosion, and progressive fibrosis following minor friction (1, 2). Beyond acute lesions, patients often suffer from chronic wound healing impairment. Recurrent inflammation and fibrotic responses ultimately lead to hand deformities such as pseudo-syndactyly and joint contractures, severely compromising limb function and quality of life (3). Although emerging technologies like CRISPR/Cas9 gene editing offer hope for a definitive cure (4), current clinically available therapies remain primarily supportive and wound-directed rather than curative. Importantly, recent FDA-approved therapies such as beremagene geperpavec-svdt (VYJUVEK), a topical gene therapy delivering *COL7A1*via HSV-1 vector, and prademagene zamikeracel (ZEVASKYN), an autologous gene-corrected keratinocyte sheet therapy, have expanded treatment options for localized wound management in RDEB patients (5–7). However, surgical management of established contractures remains necessary.

Currently, Vaseline gauze is commonly used postoperatively for hand wounds (8). However, this traditional dressing tends to adhere firmly to fragile neo-epithelium during removal, causing mechanical stripping and pain. This not only negates the benefits of surgical release but may also induce refractory wounds due to secondary injury. Therefore, there is an urgent need for materials that combine antimicrobial properties, non-adhesion, and comfort. Silver Sulfadiazine (SSD) has demonstrated excellent antibacterial effects and wound healing promotion across various skin injuries (9). Based on the antimicrobial and non-adherent properties of SSD, this study introduced SSD dressings to replace traditional Vaseline gauze, combined with compression therapy using soft brace (8). We aimed to evaluate the association between this SSD-modified soft brace strategy and perioperative outcomes and long-term home compliance.

## 2 Materials and methods

### 2.1 Study subjects and grouping

We conducted a retrospective cohort study of patients with genetically confirmed recessive dystrophic epidermolysis bullosa (RDEB) who underwent surgical release of hand scar contractures at our institution between January 2017 and December 2023. Eligible participants were aged 8–21 years and presented with metacarpophalangeal (MCP) hyperextension or interphalangeal (IP) flexion deformities necessitating surgical intervention. Patients with sulfonamide allergies, severe cardiac/hepatic/renal comorbidities precluding surgery, or incomplete clinical records were excluded. Ultimately, 35 patients met the inclusion criteria and were allocated to two groups based on the postoperative dressing regimen: seventeen patients treated from January 2017 to January 2020 received standard Vaseline gauze (Traditional Group), while eighteen patients treated from February 2020 to December 2023 received a modified silver sulfadiazine (SSD) oil-based dressing (Modified Group). Baseline nutritional status (preoperative hemoglobin and albumin levels), unilateral/bilateral surgery, number of fingers released, and history of previous hand surgery were collected to document comparability (Table 1). The study protocol was approved by the Hospital Ethics Committee, and written informed consent was obtained from all legal guardians prior to data collection.

**Table 1:** Baseline Characteristics and Perioperative Indicators.

Variable	Traditional(*n* = 17)	Modified(*n* = 18)	*P*-value
Age (y), Mean ± SD​	14.18 ± 4.11​	14.33 ± 3.88​	>0.05​
Median [min, max]	14.00 [8.00, 21.00]	14.50 [8.00, 21.00]	
Gender, *n* (%)​			>0.05​
M	8 (47.1%)	9 (50.0%)	
F	9 (52.9%)	9 (50.0%)	
Previous hand surgery, *n* (%)​			>0.05​
no	16 (94.1%)	16 (88.9%)	
yes	1 (5.9%)	2 (11.1%)	
Preoperative Albumin (g/L), Mean ± SD	38.43 ± 2.43	38.00 ± 2.89	>0.05
Glucose(mmoml/L)	4.99 ± 0.25	5.02 ± 0.55	0.79
Preoperative Hemoglobin (g/L), Mean ± SD	103.63 ± 3.35	101.61 ± 4.53	0.16
Unilateral/Bilateral surgery, n	0/17	0/18	>0.05
Number of fingers released	10	10	-
Dressing Changes (n)​	4.18 ± 0.73​	2.33 ± 0.49​	8.60E-07*
Anesthesia Time per change (min)​	53.71 ± 5.89​	27.83 ± 3.91​	4.80E-07*
Blood loss per change (mL)​	81.76 ± 12.86​	61.67 ± 12.95​	8.9E-5*
Epithelial Tear (Stripping), *n* (%)​			1.6E-3​
no	6 (35.3%)	16 (88.9%)	
yes	11 (64.7%)	2 (11.1%)	
Time to wound healing (days)	36.24 ± 5.09	23.33 ± 3.40	＜0.001

### 2.2 Intervention protocol

All surgeries were performed by the same team using standardized scar release techniques. Both groups wore customized soft brace (8) postoperatively for compression fixation, ensuring no compromise to distal circulation. The soft brace was custom-fabricated from low-durometer thermoplastic material with hook-and-loop straps, providing consistent compression; the exact compression pressure was titrated to maintain distal perfusion. K-wires were placed to immobilize digits in full extension and were typically removed at 3–4 weeks once stable epithelialization was confirmed. Pin-site care was performed weekly using the same assigned dressing regimen; no routine prophylactic systemic antibiotics were administered. Postoperative analgesia included scheduled oral non-steroidal anti-inflammatory drugs and rescue opioids as needed. No split-thickness skin grafts or bioengineered skin substitutes were applied; all wounds healed by secondary intention. Beyond the composition of the oil-based dressing, all other perioperative protocols were strictly standardized. Following scar contracture release, pseudosyndactyly was completely dissected and contracted bands were meticulously released until full metacarpophalangeal (MCP) joint extension was achieved. Subsequent immobilization was maintained via K-wire fixation of the digits in a fully extended position. The resultant wound beds were then dressed with a single layer of the assigned oil-based dressing, followed by layered coverage with sterile gauze and elastic bandages. This standardized wrapping technique yielded a uniform, bulky dressing configuration resembling a boxing glove, ensuring consistent compression and protection across all subjects.

Traditional Group: Wounds were covered with Vaseline gauze and sterile dry gauze. Dressing changes were performed under general anesthesia every 7 days until complete epithelialization.

Modified Group: Wounds were covered with URGO SSD (1% silver sulfadiazine lipido-colloid) dressings, which provide broad-spectrum antimicrobial effects and non-adherent lipidocolloid technology. Dressing changes were performed every 7 days until healing.

Both groups were instructed to continue wearing the braces for ≥12 h daily after discharge and to attend regular follow-ups.

### 2.3 Outcome measures

#### 2.3.1 Perioperative indicators

Total number of dressing changes, duration of each change (under anesthesia), rate of epithelial stripping (defined as visible detachment of neoepithelium during dressing removal, assessed clinically by the attending surgeon), incidence of refractory wounds (failure of epithelialization within 4 weeks or need for re-intervention), and estimated blood loss during dressing changes (quantified by weighing surgical gauze before and after the procedure, with 1 g approximating 1 mL blood loss). Wound healing duration was defined as the time from surgery to hospital discharge, at which point the attending surgeon clinically confirmed complete epithelialization without residual erosions.

#### 2.3.2 RDEB home care compliance scale (RHCS)

Due to the lack of standardized instruments for assessing RDEB-specific behavioral management, we developed the RHCS. This scale is exploratory and has not been externally validated. It is a 100-point scale comprising three domains assessed by clinicians based on structured interviews with patients/parents and direct observation during follow-up. The detailed scoring rubric is provided in Supplementary Table S1 and summarized as follows:

1. Brace Wearing Behavior (0–40 points): Evaluated based on the average daily duration of brace wear (<6 h = 0 pts; 6–8 h = 10 pts; 8–10 h = 20 pts; 10–12 h = 30 pts; ≥12 h or as prescribed = 40 pts).
2. Pain & Discomfort Tolerance (0–35 points): Assessed exclusively using the Wong-Baker FACES Scale (0 = “hurts most”, 10 = “no hurt”) across three dimensions: (i) Resting pain over the past week, converted to a 0–15 point scale (15 = no hurt, 0 = worst pain); (ii) Discomfort during brace handling and wear, scored 0–10 points; (iii) Nighttime awakening due to pain or spontaneous blisters, scored 0–10 points. The sum constitutes the 35-point domain.
3. Daily Maintenance Cooperation (0–25 points): Assessed by willingness to undergo daily wound/brace inspection (0–10 pts), adherence to hygiene and brace maintenance (0–10 pts), and proactive reporting of new lesions to clinicians (0–5 pts).

Scores for each of the three domains are reported separately in the results to avoid over-interpreting the total score, as the Pain & Discomfort domain may partially capture the direct treatment effect of the non-adherent dressing. Higher cumulative scores indicate better compliance. Internal consistency reliability of the RHCS was evaluated using Cronbach's *α* coefficient.

#### 2.3.3 Long-term follow-up

Follow-up was initiated from the date of surgery and continued until the occurrence of hand scar contracture recurrence (defined as web-space re-syndactyly or finger re-adhesion preventing functional hand opening, or need for repeat surgical intervention). Recurrence time was recorded. The monthly incidence of spontaneous nocturnal blisters (defined as new blisters arising between 22:00 and 06:00, documented by caregivers in a daily diary and verified at outpatient visits) was also documented. All patients underwent uniform, continuous follow-up at regular intervals (every month) after discharge.

### 2.4 Statistical analysis

Statistical analyses were performed using SPSS version 27.0 and the Sangerbox online platform (http://vip.sangerbox.com) (10). Continuous variables are expressed as mean ± SD and compared using independent sample t-tests or Mann–Whitney U tests where appropriate. Categorical variables were analyzed using Chi-square or Fisher's exact tests. Kaplan–Meier survival analysis with log-rank test was used for recurrence evaluation. Univariable Cox regression was used to calculate the hazard ratio. The *P*-value reported for recurrence comparison corresponds to the log-rank test, while the HR and 95% CI are derived from the univariable Cox model. *P* < 0.05 was considered statistically significant. Internal consistency reliability of the RHCS was evaluated using Cronbach's *α* coefficient.

## 3 Results

### 3.1 Baseline characteristics and scale reliability

No significant differences were observed in baseline characteristics between groups (Table 1), though absence of statistical significance in a sample of 35 does not definitively prove comparability. Reliability analysis showed the RHCS scale had an excellent overall Cronbach's *α* of 0.884 (standardized *α* = 0.883).

### 3.2 Comparison of perioperative efficacy

The Modified Group showed favorable results compared to the Traditional Group in all perioperative indicators (Table 1). The average number of dressing changes in the Modified Group was 2.33 ± 0.49, representing a ∼44% reduction compared to the Traditional Group (4.18 ± 0.73, *P* < 0.001), with a nearly 50% reduction in anesthesia time per change (27.83 ± 3.91 min vs. 53.71 ± 5.89 min, *P* < 0.001). Average blood loss during dressing changes was also significantly lower in the Modified Group (61.67 ± 12.95 mL vs. 81.76 ± 12.86 mL, *P* < 0.001). Time to wound healing (defined as days from surgery to complete epithelialization at discharge) was significantly shorter in the Modified Group (23.33 ± 3.40 days vs. 36.24 ± 5.09 days in the Traditional Group, *P* < 0.001). This difference may be attributable to the non-adhesive property of the SSD dressing, as the rate of epithelial stripping was significantly reduced [11/17 [64.7%] vs. 2/18 [11.1%], *P* = 1.6E-3], and no refractory wounds occurred in the Modified Group. No allergic reactions such as contact dermatitis were observed in either group of patients.

### 3.3 Home care compliance and long-term recurrence

Domain-specific RHCS scores (Table 2) showed that the Modified Group achieved significantly higher scores across all three domains—Brace Wearing Behavior (32.11 ± 5.10 vs. 19.24 ± 5.93, *P* < 0.001), Pain & Discomfort Tolerance (30.39 ± 4.64 vs. 14.65 ± 7.68, *P* < 0.001), and Daily Maintenance Cooperation (22.44 ± 3.71 vs. 12.24 ± 9.36, *P* = 5.1E-4)—with the largest absolute improvement in the Brace Wearing Behavior domain. The total RHCS score was 84.94 ± 9.62 in the Modified Group, significantly higher than 46.12 ± 19.12 in the Traditional Group (*P* < 0.001). This improvement is primarily attributed to the substantially lower incidence of spontaneous nocturnal blisters [2/18 [11.1%] vs. 11/17 [64.7%], *P* = 1.6E-3] and the minimal adherence of the SSD dressing to the wound bed, though the total score should be interpreted cautiously as it includes pain measures directly affected by the intervention.

**Table 2:** RHCS domain scores, long-term outcomes and follow-up.

Variable	Traditional (*n* = 17)	Modified (*n* = 18)	*P*-value
RHCS_Behavior Score (0–40)	19.24 ± 5.93	32.11 ± 5.10	8.7e-6
RHCS_Tolerance Score (0–35)	14.65 ± 7.68	30.39 ± 4.64	2.9e-6
RHCS_Maintenance Score (0–25)	12.24 ± 9.36	22.44 ± 3.71	5.1e-4
RHCS – Total Score (0–100)​	46.12 ± 19.12​	84.94 ± 9.62​	1.1E-6*
Refractory Wound, *n* (%)​			7.6E-03​
no	11 (64.7%)	18 (100%)	
yes	6 (35.3%)	0 (0%)	
Night Blisters, *n* (%)​			1.6E-003​
no	6 (35.3%)	16 (88.9%)	
yes	11 (64.7%)	2 (11.1%)	
Mean time-to-event or last follow-up (months), Mean ± SD	31.61 ± 2.28	28.47 ± 2.70	2.3e-3
Censored, *n* (%)	6 (35.3)	10 (55.6)	-
Recurrence events, *n* (%)	11 (64.7%)	8 (44.4%)	-

a *Mean time-to-event includes time to recurrence for patients who experienced recurrence and time to last follow-up for censored patients.*

Univariable Cox regression analysis revealed that treatment with the modified SSD dressing was associated with delayed recurrence (HR 0.39, 95% CI 0.16–0.95; *P* = 0.03 from log-rank test) (Supplementary Figure 1). The confidence intervals were wide and the number at risk beyond 30 months was small (only 1–2 patients), thus the tail of the curve is unstable and these results should be interpreted cautiously. During follow-up, recurrence occurred in 11 of 17 patients (64.7%) in the Traditional group and 8 of 18 (44.4%) in the Modified group; the remaining 6 and 10 patients were censored, respectively.

## 4 Discussion

The management of hand scar contractures in patients with RDEB is a protracted, painful, and often cyclical process. This study focused on the modification of perioperative soft brace and provides evidence suggesting potential clinical benefits associated with the modified SSD soft brace regimen.

Our findings demonstrate that, compared to traditional petrolatum dressings, the modified SSD lipido-colloid dressing was associated with​ multidimensional advantages in the postoperative management of RDEB hands. From a perioperative perspective, the significant reduction in both the frequency and duration of dressing changes may reduce the risks and burdens associated with repeated general anesthesia. While SSD dressings are conventionally utilized in burn care for their excellent antimicrobial and wound protection properties, their application here was associated with reduced dressing-related trauma. This efficacy is primarily attributed to the unique physical properties of the SSD dressing: its lipido-colloid matrix establishes a moist, non-adherent healing environment, likely helps avoid the mechanical trauma to neo-granulation tissue typically induced by traditional dressing removal (11).

Furthermore, RDEB wounds are highly susceptible to colonization by pathogens such as *Staphylococcus aureus (*12). The sustained release of silver ions from the SSD dressing provided broad-spectrum antimicrobial protection, which may have contributed to reduced wound complications, including the absence of refractory wounds in the Modified Group, ensuring smooth epithelialization.

More importantly, the enhancement of dressing comfort appeared to correlate with improved long-term reported tolerance. We observed that patients in the Modified Group exhibited significantly higher home care compliance scores, particularly in the Pain & Discomfort domain, while Brace Wearing Behavior also improved independently. This correlates strongly with the drastic reduction in spontaneous nocturnal blistering. For RDEB patients, the skin is exquisitely fragile; even minimal friction can trigger new bullae, leading to intense pain and psychological aversion that manifests as resistance to brace wear. In contrast, the SSD dressing, owing to its non-adherent and antimicrobial nature, minimized epidermal stripping and secondary damage during both clinical changes and home care. When patients experienced less pain, their willingness to comply with brace wear may have increased, thereby helping to maintain surgical correction. However, the total RHCS score should not be overinterpreted as a validated independent measure of adherence, given that pain relief is an intrinsic component of the intervention effect.

The longer recurrence interval observed in the Modified Group suggests that optimized local wound care may be associated with prolonged postoperative functional maintenance, although causality cannot be confirmed in this retrospective, non-randomized, sequential cohort study.

Several limitations must be acknowledged. As a single-center retrospective analysis, this study is constrained by a relatively small sample size and a historical cohort design, which introduces the possibility of temporal confounding (e.g., changes in perioperative care over time beyond the dressing type). The manuscript now uses cautious language to avoid implying causation. The RHCS scale remains exploratory and lacks external validation; domain-specific scores are reported to mitigate overinterpretation. The Kaplan–Meier curves had limited power beyond 30 months, with very few patients at risk at the latest time points, and the wide confidence intervals of the hazard ratio should be emphasized. Measurement bias is possible as outcome assessors were not blinded. Finally, certain baseline variables such as validated EB severity scores (e.g., BSCS/BCS), preoperative contracture angles, wound area, and hand function metrics were not uniformly available and could not be fully adjusted for.

Looking forward, as our understanding of RDEB pathogenesis deepens, targeted pharmacotherapies (such as TGF-*β* signaling modulators like Losartan (13) are expected to synergize with optimized local care strategies. Concurrently, a multidisciplinary team (MDT) approach will remain indispensable in balancing surgical reconstruction, rehabilitative nursing, and genetic counseling (14, 15).

In conclusion, in this retrospective cohort, the SSD-containing non-adherent dressing used with a soft brace was associated with fewer dressing changes, less dressing-related trauma, and better reported tolerance. Prospective controlled studies with validated functional and compliance outcomes are required.

## Data Availability

The original contributions presented in the study are included in the article/Supplementary Material, further inquiries can be directed to the corresponding author.

## Supplementary material

Supplementary Figure S1 The Kaplan–Meier analysis.

Supplementary Table S1 Detailed Scoring Rubric for the RDEB Home Care Compliance Scale (RHCS).
